# Influence of Different Protocols on In‐Office Bleaching: Whiteness Difference and Hydrogen Peroxide Penetration

**DOI:** 10.1111/jerd.13501

**Published:** 2025-06-25

**Authors:** Deisy Cristina Ferreira Cordeiro, Gabrielle Gomes Centenaro, Michael Willian Favoreto, Maria Alice de Matos Rodrigues, Alessandra Reis, Alessandro Dourado Loguercio

**Affiliations:** ^1^ Department of Restorative Dentistry State University of Ponta Grossa Paraná Brazil; ^2^ Department of Restorative Dentistry, School of Dentistry Tuiuti University of Paraná Paraná Brazil

**Keywords:** dental enamel permeability, hydrogen peroxide, tooth bleaching, tooth permeability

## Abstract

**Objectives:**

To evaluate whiteness difference (WI_D_) and hydrogen peroxide (HP) penetration into the pulp chamber in extracted teeth subjected to various in‐office application protocols.

**Materials and Methods:**

A total of 154 human premolars were randomly assigned to 11 groups (*n* = 14). The groups were divided into: Pola Office+ and Pola Rapid (SDI), and the following protocols: three applications of 8 min (3 × 8), two applications of 8 min (2 × 8), one application of 8 min (1 × 8), one application of 16 min (1 × 16), and one application of 24 min (1 × 24). These protocols were selected based on manufacturer recommendations and the aim of exploring simplified and alternative approaches applicable to clinical practice. One group served as a negative control. WI_D_ was assessed using a digital spectrophotometer before and after two sessions. HP concentration in the pulp chamber was measured using UV–Vis spectrophotometry. ANOVA two‐way and Tukey's and Dunnett's tests were used for data analysis.

**Results:**

Significant WI_D_ were observed after two sessions, with greater changes in the 2 × 8, 3 × 8, and 1 × 24 protocols (*p* < 0.001). Lower levels of peroxide penetration were found with shorter application, such as the 1 × 8 protocol, while higher levels were detected in the 3 × 8 protocol (*p* < 0.0001).

**Conclusion:**

Among the evaluated protocols, the 2 × 8‐min and 1 × 24‐min applications provided the best balance between achieving significant whitening and minimizing hydrogen peroxide penetration into the pulp chamber, making them effective and safer alternatives for in‐office bleaching.

**Clinical Relevance:**

Shorter application times (2 × 8 min) or a single prolonged application (1 × 24 min) provide effective alternatives for achieving excellent whitening index results. These protocols reduce hydrogen peroxide penetration into the pulp chamber while also shortening the overall procedure time, enhancing both safety and efficiency.

## 1 Introduction

Concern for aesthetics in general has also been reflected in the field of dentistry, as the quest for the perfect smile has increased the demand for tooth bleaching, making it one of the most requested procedures by patients seeking a whiter and more perfect smile [1]. Among the bleaching techniques, the in‐office approach uses high concentrations of hydrogen peroxide, which allows for a shorter treatment time compared to at‐home bleaching [2, 3].

Consequently, a significant percentage of patients typically experience tooth sensitivity induced by tooth bleaching when in‐office bleaching gels are applied [2, 3]. A literature review showed that about 60% of patients reported tooth sensitivity after in‐office bleaching [3], which is the most common adverse effect of this procedure [4, 5, 6, 7]. It is accepted that bleaching‐induced tooth sensitivity occurs because hydrogen peroxide, upon reaching the pulp, produces an inflammatory reaction, with the release of inflammatory mediators responsible for local vasodilation, increased vascular permeability [8, 9, 10], and mild to moderate tooth sensitivity [5, 6, 7].

To mitigate this side effect caused by bleaching products, various therapies have been suggested, including the application of desensitizing agents [11, 12, 13, 14], the administration of analgesics or anti‐inflammatory drugs [15, 16, 17], the use of bleaching gels with desensitizing agents [18, 19], or the use of in‐office bleaching gels with lower hydrogen peroxide concentrations [20, 21, 22, 23]. However, reducing the contact time or the frequency of bleaching gel applications appears to be a straightforward and effective approach to decreasing the amount of hydrogen peroxide inside the pulp chamber [24, 25, 26]. Previous studies have suggested that varying exposure times and the number of bleaching gel applications may optimize both the safety and efficacy of in‐office bleaching procedures. For example, studies [5, 25, 26, 27] have shown that shorter exposure times, such as 10–15 min, can reduce hydrogen peroxide penetration into the pulp chamber while maintaining an adequate bleaching effect. Additionally, modifying the number of applications during a bleaching session could enhance whitening results while minimizing associated risks, such as tooth sensitivity.

For instance, studies [24, 25] showed that a lower amount of hydrogen peroxide was observed inside the pulp chamber when 10–15 min were compared to 45–60 min. Similarly, reducing the number of renewals of bleaching gels at each in‐office bleaching session leads to a lower amount of hydrogen peroxide in the pulp chamber [25, 26]. The reasons some manufacturers indicate changes in the gel during the application protocol is due to the peroxide decomposition and the pH reduction. Over time, the decomposition of some in‐office bleaching gel [25, 27, 28, 29] tends to lower the pH [27, 30, 31], which can potentially provoke several changes in the chemical composition, morphology, and mechanical properties of the dental structure [32, 33, 34].

On the other hand, this decreases in the amount of hydrogen peroxide available, whether by reducing the contact time or the number of renewals of bleaching gel applications, can impact the bleaching efficacy [5, 25, 26, 27]. For example, studies [25, 26] showed that when in‐office bleaching gel was applied for 5–15 min, a lower whitening effect was observed compared to 30–45 min of application. Indeed, it is not clear in the previous literature, which is the minimal application time or number of bleaching gel applications to achieve a higher bleaching efficacy, without lower risk of hydrogen peroxide inside the pulp chamber, and consequently, less tooth sensitivity.

Therefore, the objective of this in vitro study is to evaluate the whitening effect and the penetration of hydrogen peroxide into the pulp chamber of human teeth subjected to different application protocols (number of changes/application time) with two in‐office bleaching gels. The selected exposure times and application protocols were based on common clinical practices and existing literature. Shorter application times (1 × 8 min) and longer durations (1 × 24 min) were chosen to simulate typical clinical scenarios. Additionally, the 2 × 8 and 3 × 8‐min protocols represent multiple short‐duration applications, frequently used in‐office. Protocols with longer exposure times, such as 2 × 16 or 3 × 16 min, were not included, as previous studies suggest that extended exposure does not substantially improve efficacy and may increase the risk of hydrogen peroxide penetration into the pulp [5, 25, 26, 27]. The hypotheses of this study are that significant differences in whitening index difference (WI_D_) and hydrogen peroxide penetration into the pulp chamber will be observed when comparing (1) different application protocols, (2) the number of treatment sessions, and (3) types of in‐office bleaching agents. These differences are expected because varying application times and protocols can influence the effectiveness of bleaching and the extent of hydrogen peroxide diffusion into the pulp chamber, which is directly related to tooth sensitivity and bleaching efficacy.

## 2 Materials and Methods

### 2.1 Ethical Approval and Criteria for Teeth Selection

This study was approved by the Ethics Committee of the State University of Ponta Grossa (Ponta Grossa/PR/Brazil) under protocol number 6.782.857. One hundred and fifty‐four first and second healthy premolars were obtained from the Human Teeth Local Bank. They were evaluated using a 10× magnification microscope (Lambda LEB‐3, ATTO instruments, Hong Kong, China), and only those without superficial morphological changes or enamel cracks were included. The selected teeth were required to have a baseline whiteness index for dentistry (WI_D_) [35] 15 units or smaller.

Premolars were selected due to their higher availability, as they are often extracted for orthodontic purposes. Additionally, their enamel and dentin thicknesses are representative of clinical conditions, making them suitable for assessing hydrogen peroxide diffusion and whitening changes, as demonstrated in several previous studies [30, 31]. Before and during the bleaching period, the specimens were immersed in artificial saliva (Efficacy Pharmacy, Ponta Grossa, PR, Brazil). Additional details regarding its composition and use are provided in the final color evaluation section.

### 2.2 Simple Size Estimation

The primary outcome of this study was the whitening difference in WI_D_. Based on a previous study [36], the whitening difference of teeth subjected to in‐office bleaching with 37.5% hydrogen peroxide Pola Office+ (SDI, Victoria, Australia) was 6.9 ± 1.8 units of WI_D_. In a superiority test, using a two‐sided test with an alpha of 5% and a study power of 80%, 11 teeth for the group were needed to detect a difference of 2.6 units of WI_D_ that is perceptible to the human eye [37]. Three teeth were added to each group, accounting for possible losses. Therefore, the final sample size was 14 teeth per group.

### 2.3 Experimental Groups and Randomization

The teeth were randomized using a blocked randomization sequence generated in Microsoft Excel and distributed into 11 groups (*n* = 14). One group was not exposed to bleaching agents and served as the negative control. The remaining groups were categorized according to the bleaching gel used: (1) Pola Office+ (SDI, Victoria, Australia) [38] and Pola Rapid (SDI, Victoria, Australia) [39], and the following protocols: (2) three applications of 8 min (3 × 8), two applications of 8 min (2 × 8), one application of 8 min (1 × 8), one application of 16 min (1 × 16), and one application of 24 min (1 × 24). To ensure allocation concealment, the randomization sequence was generated by an investigator not involved in any treatment or outcome assessments. This sequence was concealed until immediately before treatment allocation. Due to the nature of the interventions, the investigator performing the treatments could not be blinded to group assignment. However, the investigator responsible for all outcome assessments, including hydrogen peroxide penetration and whitening measurements, was blinded to group allocation to minimize the risk of measurement bias.

### 2.4 Initial and Final Concentrations of Hydrogen Peroxide in the Bleaching Agents

The bleaching gels used in this study were titrated with a standardized potassium permanganate solution prior to the bleaching procedure, as described in previous studies [40, 41]. This titration aimed to determine the initial concentrations of the active agent in the gel and verify their consistency with the manufacturers' specifications. Additionally, the same measurements were performed after 8, 16, and 24 min to assess the stability of the bleaching agents over time. All analyses were conducted in triplicate to ensure accuracy and reproducibility.

### 2.5 Viscosity of the Bleaching Gel

The viscosity of the bleaching gels was measured based on their shear rate using a controlled strain rheometer (DHR‐2, TA Industries, Newcastle, DE, USA) with a 2° cone plate geometry of 40 mm diameter [42]. The rheometer was equipped with a Peltier accessory integrated with a heating/cooling system, ensuring a consistent sample temperature of 37°C [43], which is equivalent to the temperature of the mouth. All tests were conducted at this constant temperature to evaluate the thixotropic behavior of the gels, subjecting them to a continuous flow during the recommended application time for each group at a constant shear rate of 5 s^−1^. Analyses were performed in triplicate to ensure precision and consistency of the results [11].

### 2.6 Specimen Preparation and Randomization

Selected teeth were assigned numbers, and a simple randomization process was conducted using an Excel spreadsheet. The roots were cutted employing the Isomet 1000 equipment (Buehler Ltd., Lake Bluff, USA) ensuring approximately three millimeters from the enamel–cement junction in the apical direction. Subsequently, the pulp tissue was meticulously removed, and the access to the pulp cavity was slightly widened using a spherical drill. Special care was taken to avoid contact with the inner buccal region of the pulp cavity. Amplification is required to accommodate approximately 25 μL of acetate buffer solution, introduced into the pulp chamber via a micropipette during permeability assessment.

### 2.7 Evaluation of the Specimen Thickness

**FIGURE 1:**
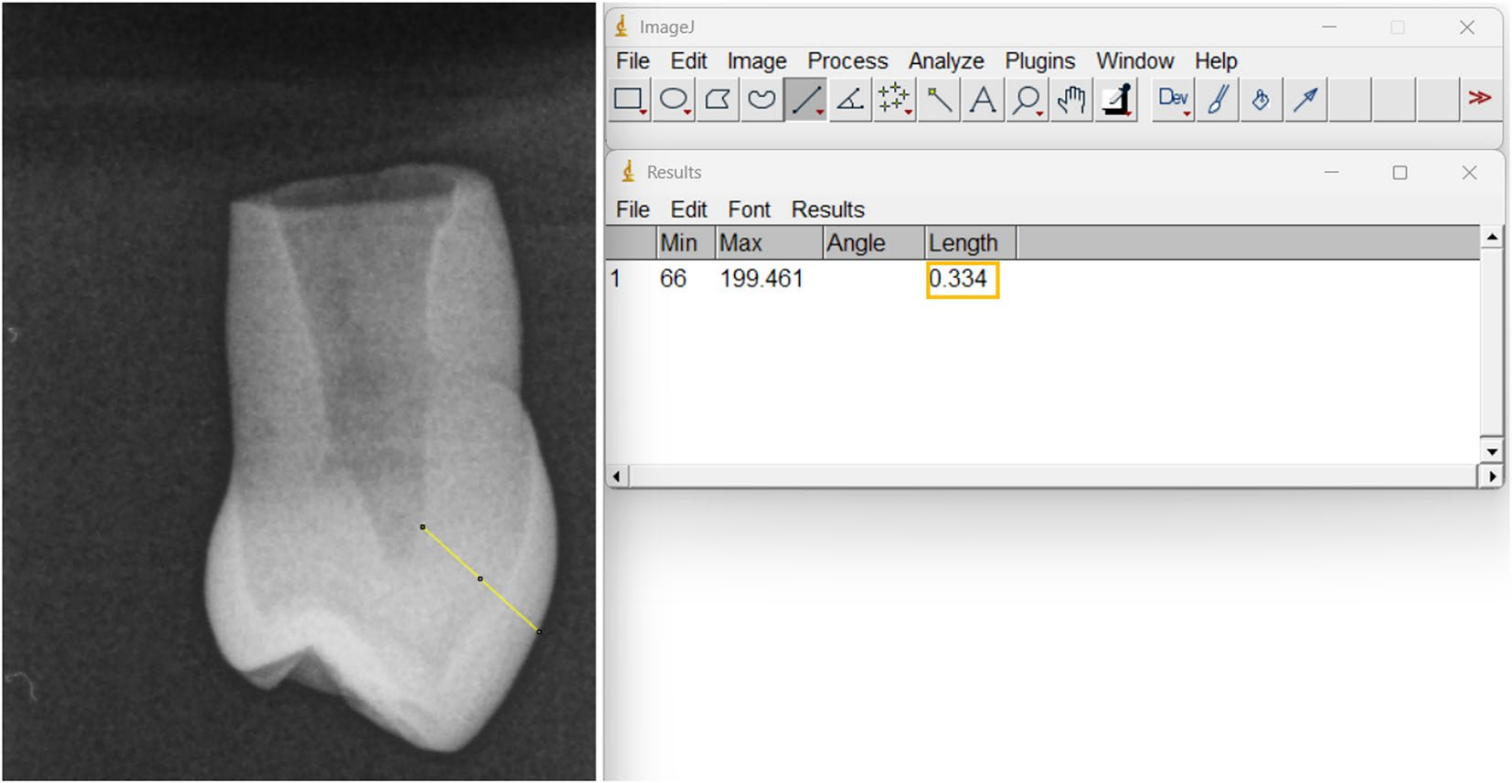
Measurement of buccal thickness in a premolar tooth. The left panel shows the digitized radiographic image, with the buccal thickness indicated along the white reference line. The right panel displays the corresponding measurement value, expressed in centimeters (cm).

Following the preparation of specimens, X‐ray radiographs were taken using the Timex 70C equipment (Gnatus, Ribeirão Preto, SP, Brazil) [44]. To capture these radiographs, each tooth's mesial face was placed in contact with the X‐ray film. A standardized exposure time of 0.5 s was maintained, with a 30‐cm focus‐object distance. The central X‐ray beam (70 kVp; 7 mA) was positioned at a 90° angle to the tooth's distal surface. Post‐exposure, the radiographic images were digitally acquired using the free ImageJ software (National Institutes of Health, USA). To ensure a consistent and standardized sample for the study, only teeth with dentin and enamel thicknesses between 2.5 and 3.5 mm were included. Dentin thickness was measured using the same software employed for image acquisition (ImageJ software, National Institutes of Health, USA; Figure 1). The teeth were positioned laterally, and the measurement was taken from the outermost part of the vestibular surface to the pulp horn to ensure a consistent and standardized sample for the study.

### 2.8 Baseline Whiteness Measurement

To standardize the position of the spectrophotometer, individual impressions were made using a green dense silicone paste (Coltoflax and Perfil Cub Kit, Vigodent, Rio de Janeiro, RJ, Brazil) [45]. An orifice of 6 mm diameter was cut in the middle third of the buccal surface of each green silicone guide to allow the insertion of the tip of the spectrophotometer [40]. Initial color parameters (L*, a*, and b*) were measured with a digital spectrophotometer (VITA Easyshade Advance 4.0, VITA Zahnfabrik, Bad Säckingen, Germany) before the treatments began. Before each measurement, the spectrophotometer was calibrated according to the manufacturer's guidelines to ensure accuracy and prevent any instrumental errors.

The L* value signifies lightness, ranging from 0 (black) to 100 (white). The a* value indicates color along the red–green axis, while the b* value represents color along the yellow–blue axis. The WI_D_ was calculated using the formula: WI_D_ = 0.511L* − 2.324a* − 1:100b* [35]. To ensure consistency in color measurements, all assessments were performed by a single operator using a digital spectrophotometer (VITA Easyshade Advance 4.0; Vita Zahnfabrik, Bad Säckinger, Germany). This approach was chosen to minimize variability and ensure that all readings were obtained under standardized conditions.

### 2.9 Obtaining the Analytical Curve of Concentrations Hydrogen Peroxide

The study employed analytical products directly without prior purification, and all solutions were prepared using deionized water. Initially, a standard analytical curve of concentrations of hydrogen peroxide was established from a stock solution of 5.000 μg mL^−1^ of hydrogen peroxide. This stock solution was prepared from a concentrated solution obtained from a local pharmacy (Efficacy Pharmacy, Ponta Grossa, PR, Brazil), which was then diluted in an acetate buffer solution (pH = 4) and titrated by conventional methods. The solution underwent titration with a potassium permanganate solution to determine both its analytical grade and actual concentration of hydrogen peroxide [44].

Based on this hydrogen peroxide initial concentration, serial volumetric dilutions ranging from 0.000 to 0.397 μg mL^−1^ were performed to construct the hydrogen peroxide analytical curve. After that, hydrogen peroxide concentrations were measured using a Cary UV–Vis 100 spectrophotometer (Varian, Palo Alto, CA, USA). This process of obtaining the analytical curve of hydrogen peroxide concentrations established a standard reference line used to extrapolate the results of the study samples, with an excellent correlation coefficient of *R* = 0.999 (specific data not reported).

### 2.10 Bleaching Protocol and Hydrogen Peroxide Penetration Into Pulp Chamber

In all groups, the specimens were vertically fixed to a wax plate with the occlusal surface facing the plate. Prior to the application of the bleaching agent, the buccal surface of each specimen was isolated by applying a light‐cured resin barrier (Gingival Barrier, SDI, Victoria, Australia), forming an enclosure of 6 × 6 mm. A 25‐μL aliquot of acetate buffer (pH = 4) was introduced into the pulp cavity of each tooth to retain all hydrogen peroxide that entered the pulp cavity during the bleaching procedures. A single experienced operator handled the application of the materials. With the self‐mixing tip properly attached to the double‐barrel syringe containing the bleaching gel, the piston was slowly pressed to allow the peroxide and thickening agent to mix. A small amount of the gel was dispensed into a container before application to the tooth surface, ensuring that the product was thoroughly homogenized before use.

Following the mixing process, the bleaching agent was applied in the buccal enamel area according with different experimental groups. The application of bleaching gels continued until the buccal area of the designated teeth was entirely covered. After the predetermined time and number of applications for each group, the bleaching gel was removed using gauze and meticulously rinsed with deionized water. Each group underwent two bleaching sessions with a 1‐week interval between them.

Following this, the acetate buffer solution within the pulp cavity of each tooth was removed using a mechanical micropipette and transferred to a glass tube. This process involved rinsing the pulp cavity of each tooth four times with 25 μL of acetate buffer, and each rinse was then transferred to the same glass tube. Thereafter, 2.725 μL distilled water was introduced into the glass tube along with 100 μL of 0.5 mg/mL Leucocrystal Violet (SigmaChemical Co, St Louis, MO, USA) and 50 μL of 1 mg mL^−1^ horseradish peroxidase enzyme (Peroxidase Type VIA, Sigma Chemical Co, St. Louis, MO, USA). This sequence was repeated separately for each specimen. The resulting solution exhibited a violet color with a maximum absorbance peak at 591 nm, which was measured using a Cary100 UV–Vis spectrophotometer (Varian, Palo Alto, CA, USA). The absorbance recorded represented the highest absorption peak of the reaction between hydrogen peroxide and Leucocrystal Violet (Crystal Violet‐591 nm). In accordance with Beer's Law, absorbance correlates directly with concentration. Consequently, the concentration of hydrogen peroxide (μg mL^−1^) was determined by comparing it with the previously obtained calibration curve.

### 2.11 Final Whiteness Measurement

Subsequently, the whitening difference was assessed 48 h after the first and second bleaching sessions using a digital spectrophotometer (VITA EasyshadeAdvance 4.0, VITA Zahnfabrik, Bad Säckingen, Germany). Prior to each measurement, the device was calibrated according to the manufacturer's guidelines to ensure measurement accuracy and eliminate potential instrumental errors. During this period, the specimens were stored in artificial saliva (composed of carboxymethylcellulose, sodium chloride, potassium chloride, magnesium chloride, dibasic calcium phosphate, glycerin, xylitol, and distilled water) [46] at a controlled temperature of 37°C. The final color parameters (L*, a*, and b*) were measured after treatments. Color measurements were taken at baseline and after the first and second bleaching sessions. The whitening difference was calculated using the whiteness index for dentistry (WI_D_), according to the formula: WI_D_ = 0.511 L* − 2.324a* − 1:100b* [35]. The changes in whiteness were calculated using the following formulas: ΔWI_D_1 = WI_D after 1st session_ − WI_D baseline_ and ΔWI_D_2 = WI_D after 2nd session_ − WI_D baseline_. Perceptual and acceptable whitening changes were considered relevant when the WID exceeded the 50:50% perceptibility (WPT) thresholds (0.7 for WI_D_) and 50:50% acceptability (WAT) thresholds (2.6 for WI_D_) [37].

### 2.12 Statistical Analysis

The data were analyzed statistically, beginning with the Kolmogorov–Smirnov test to assess normality and the Bartlett test to verify the homogeneity of variances (data not shown). Given that the data met the assumptions of normal distribution and equal variances, a one‐way ANOVA was performed to analyze the thickness of the buccal teeth for each group. For the whitening difference in WI_D_ and the hydrogen peroxide concentration in the pulp chamber (measured in micrograms per milliliter), a three‐way ANOVA was employed (bleaching gel, number of changes/application time and sessions). Tukey's post hoc test was used to compare different whitening techniques. Additionally, a one‐way ANOVA followed by Dunnett's post hoc test was conducted to compare the results of the different whitening techniques with those of the control group (*α* = 0.05).

## 3 Results

### 3.1 Initial Concentrations and Degradation Over the Time of Hydrogen Peroxide in the Bleaching Agents

**FIGURE 2:**
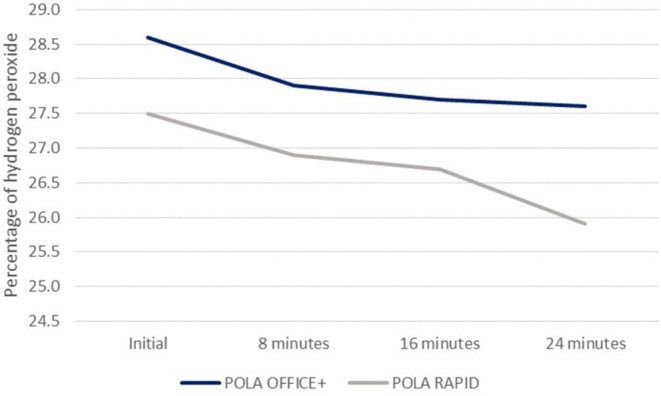
Degradation of the bleaching gels over time.

The concentration of hydrogen peroxide indicated by the manufacturer is 37.5% for the Pola Office+ bleaching agent and 38% for the Pola Rapid (before mixing) bleaching agent. However, the actual concentrations after titration were established to be 28.6% ± 0.01% and 27.5% ± 0.01%, respectively. Regarding degradation, it occurred gradually over time, but without significant concentration loss during the shorter application times. The reduction ranged between 1% and 1.6% for Pola Office+ and Pola Rapid, respectively, during the longest application period (24 min; Figure 2).

### 3.2 Viscosity of the Bleaching Gel

**FIGURE 3:**
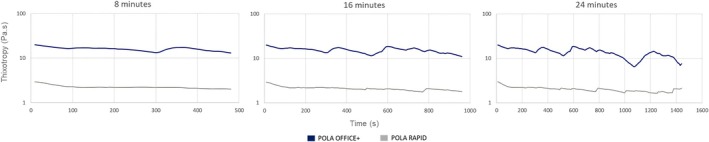
Viscosity behavior of the bleaching gels over time. Both gels demonstrated thixotropic behavior (viscosity variation over time), with Pola Rapid exhibiting less thixotropic compared to Pola Office+.

Pola Office+ is the more viscous gel and exhibits greater thixotropy, meaning its viscosity changes more significantly over time during application. In contrast, Pola Rapid becomes time‐independent after 30 s, displaying a nearly linear viscosity curve. Despite Pola Office+'s higher thixotropy, its viscosity does not decrease sufficiently by the end of the bleaching procedure to hinder its application, remaining higher than that of Pola Rapid, which maintains a relatively low viscosity throughout the process (Figure 3).

### 3.3 Tooth Specimen Characterization

The baseline whiteness, measured in WI_D_ units, was similar across all groups, ranging from 13.0 to 13.9, with no statistically significant differences between them (Table 1, *p* = 0.82). Additionally, the specimens exhibited comparable thicknesses, ranging between 3.0 and 3.5 mm, with no significant variation observed (data not shown, *p* = 0.52).

**TABLE 1:** Means and standard deviations of baseline whiteness (WI_D_ baseline) and color difference in WI_D_ in different experimental groups.

Bleaching gels	Number of changes/application time	WI_D_		
		Baseline	ΔWI_D_1^a^	ΔWI_D_2
Pola Office+	1 × 8	13.3 ± 3.1	4.4 ± 3.4 c	7.4 ± 4.0 c
	2 × 8	13.4 ± 4.1	8.0 ± 4.6 c	14.8 ± 4.2 a
	3 × 8	13.4 ± 3.7	6.4 ± 3.2 c	14.8 ± 4.1 a
	1 × 16	13.8 ± 3.5	5.3 ± 2.8 c	9.7 ± 3.6 b,c
	1 × 24	13.0 ± 3.2	9.4 ± 2.8 b,c	12.4 ± 4.2 a,b
Pola Rapid	1 × 8	13.5 ± 2.5	18.5 ± 4.1 c	20.5 ± 3.2 c
	2 × 8	13.8 ± 4.2	20.4 ± 4.9 c	25.1 ± 3.1 a,b
	3 × 8	13.1 ± 4.5	20.4 ± 4.9 c	29.7 ± 6.0 a
	1 × 16	13.9 ± 3.1	18.9 ± 3.9 c	22.8 ± 4.3 b,c
	1 × 24	13.2 ± 3.2	22.1 ± 3.9 b,c	24.1 ± 5.9 a,b

^a^ ΔWI_D_1 = WI_D after 1st session_ − WI_D baseline_; ΔWI_D_2 = WI_D after 2nd session_ − WI_D baseline_. Identical letters indicate statistically similar means (three‐way ANOVA and Tukey's test; *p* > 0.05).

### 3.4 Whiteness Index for Dentistry

Table 1 presents the means and standard deviations of the baseline WI_D_ values and whitening difference (WI_D_). All bleaching groups exhibited a highly significant and greater whitening difference compared to the control group (data not shown; *p* < 0.00000001, as determined by Dunnett's post hoc test).

Regarding whitening difference, the triple cross‐product interaction (bleaching gel, number of changes/application time, and sessions), as well as two double cross‐product interactions (bleaching gel vs. number of changes/application time and bleaching gel vs. sessions), as well as the main factor “bleaching gel” showed no statistically significant differences (Table 1, *p* > 0.21). However, a highly significant interaction was observed in one double cross‐product (number of changes/application time vs. sessions; Table 1, *p* < 0.001).

After the first session of bleaching, similar whitening differences were observed across all groups, regardless of the bleaching gel or number of changes/application time (Table 1, *p* = 0.31). However, after the second session of bleaching, highly significant differences in whitening difference emerged between experimental groups (Table 1, *p* < 0.0001). Notably, higher whitening difference was recorded for both Pola Office+ and Pola Rapid when applied in the 2 × 8, 3 × 8, or 1 × 24 protocols, compared to other groups (Table 1, *p* < 0.0001). When comparing both sessions, a highly significant whitening difference was observed only in the 3 × 8 groups, with greater change occurring in the second session (Table 1, *p* < 0.0001).

### 3.5 Hydrogen Peroxide Concentration in the Pulp Chamber

The means and standard deviations of hydrogen peroxide penetration into the pulp chamber during the bleaching protocols are presented in Table 2. All experimental groups exhibited highly significant higher hydrogen peroxide concentration in the pulp chamber compared to the control group (data not shown; *p* < 0.000001; Dunnett's post hoc test). The triple cross‐product interaction (bleaching gel, number of changes/application time, and sessions), a double cross‐product interactions (bleaching gel vs. sessions and number of changes/application time vs. sessions), as well as the main factor “sessions” showed no statistically significant differences (Table 2, *p* > 0.09). However, a highly significant interaction was observed in one double cross‐product (bleaching gel vs. number of changes/application time; Table 2, *p* < 0.0001).

**TABLE 2:** Means and standard deviations of the hydrogen peroxide concentration (μg mL^−1^) in the pulp chamber for the different gels and time protocols according to the evaluation sessions.

Bleaching gels	Number of changes/application time	First session^a^	Second session
Pola Office^+^	1 × 8	0.137 ± 0.024 a,b	0.138 ± 0.025 a,b
	2 × 8	0.240 ± 0.042 c	0.240 ± 0.043 c
	3 × 8	0.338 ± 0.031 d	0.341 ± 0.031 d
	1 × 16	0.238 ± 0.030 c	0.237 ± 0.030 c
	1 × 24	0.242 ± 0.030 c	0.242 ± 0.030 c
Pola Rapid	1 × 8	0.083 ± 0.013 a	0.083 ± 0.013 a
	2 × 8	0.163 ± 0.040 b	0.163 ± 0.040 b
	3 × 8	0.247 ± 0.024 c	0.249 ± 0.024 c
	1 × 16	0.156 ± 0.033 b	0.156 ± 0.032 b
	1 × 24	0.151 ± 0.021 b	0.151 ± 0.021 b

^a^ Identical letters indicate statistically similar means (three‐way ANOVA and Tukey's test; *p* > 0.05).

Both bleaching gels showed highly significant higher amounts of hydrogen peroxide penetration when the 3 × 8 protocol was compared to all other experimental groups. Conversely, lower amounts of hydrogen peroxide were observed for both bleaching gels in the 1 × 8 protocol compared to all other groups (Table 2, *p* < 0.0001). When comparing the two bleaching gels, Pola Office+ consistently demonstrated a higher amount of hydrogen peroxide inside the pulp chamber than Pola Rapid under the same number of changes/application times (Table 2, *p* < 0.0001).

## 4 Discussion

The results of the present study showed that after the first session, all groups exhibited similar outcomes in terms of whitening difference, regardless of the number of applications or the application time for both bleaching gels. However, after the second session, significant differences were observed regarding the number of applications, the application time, and in comparison to the first session. These findings were consistent for both bleaching gels. Based on these results, the authors accept the first and second research hypotheses and reject the third research hypothesis in terms of bleaching efficacy.

Regarding the first session, even a single 8‐min application of the bleaching agent resulted in whitening differences exceeding perceptibility and acceptability thresholds, indicating noticeable and clinically acceptable results [37]. This similarity among groups can be attributed to the high concentration of hydrogen peroxide, which, combined with the low decomposition rate demonstrated in this study and previous studies [25, 27, 28, 29], ensures rapid and effective action in terms of bleaching. These findings are particularly promising for combined bleaching protocols, where the significant initial whitening difference can be further enhanced by additional at‐home applications. Achieving such noticeable bleaching results in just 8 min underscores the efficiency of these bleaching gels in a short treatment duration. Future studies should be done to confirm this hypothesis.

However, if only in‐office bleaching was applied, the literature suggests that increasing the number of applications or multiple clinical sessions typically results in a more consistent and long‐lasting bleaching pattern [5, 25, 26, 27]. Actually, in the second session, it was observed that groups with a higher number of gel changes (2 × 8 or 3 × 8 min) exhibited a greater whitening difference compared to the same groups in the first session or with lower application time (1 × 8 min) in both sessions. This result indicates that the greater availability of the bleaching agent, provided by the changes, contributes to a more intense and significant bleaching [5, 25, 26, 27]. This is in line with the manufacturer's recommendations, which suggest that multiple applications optimize the final result.

In addition to the multiple application groups, the second session revealed that groups with longer application times (such as 3 × 8 or 1 × 24 min) demonstrated superior bleaching results. The extended contact time between the gel and the tooth surface enhances the chemical reaction necessary for effective bleaching [5, 25, 26, 27]. This allows hydrogen peroxide to penetrate deeper into the dental structure, breaking down more difficult‐to‐remove stains, resulting in superior efficacy [5, 25, 26, 27]. Therefore, performing more than one session may be a recommended strategy for patients seeking optimal results, especially when only in‐office bleaching is used.

A closer look at the groups with greater whitening difference reveals that, while the 2 × 8 min group has a shorter application time, the 1 × 24 min group uses less bleaching gel. Both factors positively contribute to reducing the cost of treatment. It is worth mentioning that, although the 3 × 8 min group also showed significant whitening difference, the longer protocol and increased use of bleaching gel reduce the advantages observed with the previous bleaching techniques. Future studies incorporating a cost–benefit analysis should be conducted to determine which technique is more economically favorable.

In addition to its efficacy, shorter protocols, such as the 2 × 8‐min session, offer a significant clinical advantage: a reduction in the application time of the bleaching gel. This benefit not only enhances the patient experience by reducing chair time and minimizing the discomfort associated with longer treatments but also optimizes dental practice efficiency. For high‐volume clinics, shorter protocols enable more patients to be treated while maintaining high‐quality results, thereby increasing overall office productivity. Moreover, by reducing both the treatment time and the amount of gel used, the procedure's cost is lowered, making whitening treatments more accessible to patients. This combination of effective results, reduced treatment time, and lower costs can significantly improve patient adherence and overall satisfaction with the procedure, underscoring the relevance of shorter‐duration protocols in clinical practice.

On the other hand, regarding the amount of hydrogen peroxide penetration into the pulp, the results of the present study showed that after the first and second sessions, all groups exhibited significant differences based on the number of applications and the application time for both bleaching gels. Additionally, a significant difference was observed when the two bleaching gels were compared. This led the authors to accept the first and third research hypotheses and to reject the second research hypothesis regarding hydrogen peroxide concentration in the pulp chamber.

Actually, after first and second sessions, the 1 × 8 min protocol showed the lowest amount of hydrogen peroxide in the pulp for both products. This reduced penetration can be attributed to the shorter application time, which limits the amount of hydrogen peroxide reaching the dental pulp when compared to protocols with more applications or extended time [24, 25, 26]. These results are particularly interesting when applied to combined bleaching protocols, as lower hydrogen peroxide penetration may lead to reduced tooth sensitivity, encouraging patients to continue the bleaching treatment. However, future studies are necessary to confirm this hypothesis.

Although it was expected that an increase in the number of applications or application time would result in higher amounts of hydrogen peroxide in the pulp, this was not observed in the present study, particularly when comparing the 3 × 8 and 1 × 24 min groups. In fact, the 3 × 8 min protocol demonstrated the highest penetration during both evaluation periods for both in‐office bleaching gels assessed. However, we acknowledge that increased hydrogen peroxide penetration could potentially lead to increased tooth sensitivity, particularly when higher concentrations of hydrogen peroxide are used. This increase can be better explained by the larger volume of gel applied rather than the longer duration of contact between the hydrogen peroxide and the tooth surface [21, 36]. The greater amount of available hydrogen peroxide appears to play a more crucial role in the penetration of the bleaching agent, in agreement with previous in vitro and in vivo studies [4, 21, 36].

The comparison of different application times for the bleaching gels reveals a complex relationship between whitening difference and permeability. The increase in the number of applications, such as 2 × 8 and 3 × 8 min, resulted in a greater whitening difference after two sessions, indicating that multiple applications and extended contact periods maximize bleaching. Additionally, longer application times, such as 1 × 24 min, while producing effective bleaching, are associated with increased peroxide penetration into the pulp, potentially leading to higher tooth sensitivity. In contrast, the 2 × 8 min protocol offers a favorable balance, providing good bleaching results with lower peroxide penetration, representing a safe and effective approach. In terms of clinical recommendations, although the 2 × 8, 3 × 8, and 1 × 24 protocols yielded the most significant whitening differences, the 2 × 8 and 1 × 24 protocols appear to offer the best balance between effectiveness and patient safety.

This is consistent with the study [7], which evaluated one of the bleaching gels used in the present study and reported similar findings in terms of bleaching efficacy and safety. Future studies should continue to explore the relationship between application times and gel properties to optimize dental bleaching strategies and ensure the best clinical outcomes.

The results of the present study showed that, when comparing both bleaching gels under the same application time, Pola Rapid exhibited lower hydrogen peroxide levels in the pulp than Pola Office+. These findings cannot be attributed to differences in hydrogen peroxide concentration, as similar concentrations were measured at both the initial and final time points, with no significant degradation observed—even after the longest application period (24 min: 1% for Pola Office+ and 1.6% for Pola Rapid). It is worth noting that a discrepancy was observed between the manufacturer's stated concentrations and the actual concentrations measured by the authors, consistent with previous reports [36]. The authors have no definitive explanation for this discrepancy. However, since both products exhibited similar peroxide concentrations in the experimental conditions, this inconsistency does not explain the differing outcomes observed between the two gels.

Instead, this difference may be attributed to variations in gel viscosity and formulation, as previously reported in the literature [42, 43]. Pola Rapid is less viscous than Pola Office+, and it could be assumed that its higher flowability might facilitate greater diffusion of hydrogen peroxide into the pulp chamber [43]. However, the opposite was observed in the present study. Two factors may help explain these results. First, while Pola Rapid maintained a consistent viscosity throughout the bleaching procedure, Pola Office+ exhibited greater thixotropy, meaning its viscosity changes more significantly over time during application. A previous study showed that increased in thixotropic behavior in in‐office bleaching gels significantly enhances the amount of hydrogen peroxide that reaches the pulp chamber [42]. Additionally, since Pola Office+ is more viscous compared to Pola Rapid, a thicker layer may form after its application, potentially affecting the amount of hydrogen peroxide that penetrates the pulp chamber [4, 21, 36].

This difference in rheological behavior may influence hydrogen peroxide penetration into the pulp chamber, as higher viscosity and greater thixotropy could reduce the diffusion rate of the bleaching agent through enamel and dentin. Future studies should be conducted to specifically evaluate whether there is a correlation between viscosity and the amount of hydrogen peroxide penetrating the dentin.

Additionally, other formulation characteristics, such as pH and additional components, may also influence hydrogen peroxide penetration. Since both in‐office bleaching gels are manufactured by the same company and are indicated for the same purposes, some differences in their bleaching compositions can be expected. Unfortunately, the exact composition of each in‐office bleaching gel is proprietary information. The Material Safety Data Sheet for Pola Office+ indicates that it contains sodium hydroxide in lower concentrations (> 0.5%), while Pola Rapid does not [38, 39]. When hydrogen peroxide is mixed with even small amounts of sodium hydroxide, an auto‐accelerating reaction occurs, significantly enhancing the decomposition of hydrogen peroxide [32]. Although this reaction did not significantly enhance the bleaching efficacy, as observed in the present study [32], it may help to explain the findings of the current study, which indicate greater penetration into the pulp chamber when Pola Office+ was used.

Although this study demonstrates that shorter protocols, such as the 1 × 8‐min session, result in less hydrogen peroxide penetration into the pulp chamber—potentially reducing tooth sensitivity—the bleaching effectiveness relies on the peroxide's ability to reach deeper areas of the dental structure. The clinical challenge lies in achieving sufficient hydrogen peroxide penetration to provide effective whitening while preventing excessive amounts from reaching the pulp and compromising its health. Protocols like the 2 × 8‐min session have shown a good balance, delivering whitening results comparable to longer protocols, with reduced peroxide penetration, which may help minimize potential clinical complications. Therefore, establishing a safe threshold for peroxide penetration is essential to ensure both the safety and effectiveness of the treatment.

Further investigations are needed to better understand how different gel formulations, variations in viscosity, and application techniques influence hydrogen peroxide penetration into the pulp. Future studies should also focus on the long‐term effects of this penetration on pulp health, as well as evaluate the impact of different clinical conditions and individual variables. Additionally, studies assessing the influence of sample storage conditions are also encouraged to ensure that the findings remain clinically relevant.

Several limitations of the present study should be noted. First, only two in‐office bleaching products were evaluated. Given the variations among the different in‐office bleaching agents available on the market [30, 31], the results may not be applicable to all such agents. Second, this is an in vitro study. While in vitro studies are crucial in dental research, providing controlled conditions for experimentation on specific variables, the absence of pulp fluids and biological responses may influence the diffusion and dynamics of the bleaching agent, potentially affecting the environmental conditions of the results. Third, this study only used sound teeth; however, we recognize the importance of evaluating teeth with different morphological alterations, such as eroded enamel. Fourth, the study did not include follow‐up assessments beyond the second bleaching session, which we acknowledge as a limitation. Future studies should incorporate follow‐up assessments to determine whether the observed whitening changes remain stable for extended periods, such as 1 week or 1 month after treatment. Therefore, future clinical trials should be conducted, particularly to evaluate the two best protocols (2 × 8‐min sessions or 1 × 24 min) identified in the present study.

## 5 Conclusions

1. The whitening difference was significantly enhanced after a second session, particularly with an increased number of applications (2 × 8 or 3 × 8) or prolonged application time (1 × 24) for both bleaching gels.
2. Notable results regarding hydrogen peroxide concentration in the pulp chamber were observed when the gel was changed twice (2 × 8) or when the application time was extended (1 × 16 or 1 × 24).

## Conflicts of Interest

The authors declare no conflicts of interest.

## Data Availability

The data that support the findings of this study are available from the corresponding author upon reasonable request.
